# 155. Antimicrobial Resistance Patters as a Predictor of Standardized Antimicrobial Administration Ratio: A National Correlation Study

**DOI:** 10.1093/ofid/ofab466.357

**Published:** 2021-12-04

**Authors:** Andrew Rubio, Mandelin Cooper, Nickie Greer, Laurel Goldin, Julia Moody, Heather Signorelli, H L Burgess

**Affiliations:** 1 HCA Healthcare/University of Tennessee, Nashville, Tennessee; 2 HCA Healthcare, Nashville, Tennessee; 3 HealthTrust Supply Chain, Nashville, TN

## Abstract

**Background:**

Data on antimicrobial usage (AU) and antimicrobial resistance (AR) is submitted to the National Healthcare Safety Network (NHSN) from facilities monthly. Bacterial proportion resistant (%R) from the AR option reports proportion of isolates resistant to specific antimicrobial categories. Standardized Antimicrobial Administration Ratio (SAAR), generated under the AU option, compares observed to predicted days of antimicrobial therapy. The purpose of this study was to evaluate the association between %R and SAAR for broad-spectrum antibacterial agents predominantly used for hospital-onset infections (BSHO) and antibacterial agents predominantly used for resistant gram-positive infections (gram-pos) in adult intensive care units (ICUs) and medical-surgical wards (M/S).

**Methods:**

This retrospective observational review utilized data reported to NHSN to examine the association of BSHO and gram-pos SAARs with %R for various phenotypic categories by quarter from 2017 through the second quarter of 2020. Phenotypic categories included methicillin-resistant Staphylococcus aureus (MRSA), vancomycin-resistant Enterococcus faecalis and faecium (VRE), extended-spectrum cephalosporin-resistant Escherichia coli and Klebsiella spp. (ESBL), and multi-drug resistant Pseudomonas aeruginosa (MDR PSA). Pearson correlations were used to quantify the associations between SAARs and %R.

**Results:**

A total of 182 institutions were included for analysis. Weak, positive correlations were observed between SAAR for BSHO in ICU and M/S for MDR PSA %R and also for ESBL %R (r = 0.14 to 0.22, all p < 0.0001). For the gram-pos SAAR in ICU and M/S, there were weak positive correlations between MRSA %R and VRE %R (r = 0.20 to 0.31, all p < 0.0001).

**Conclusion:**

SAARs are multifactorial, yet these results highlight that more resistant organisms may possibly be contributing to higher use of antimicrobials for facilities. Future SAAR calculations could consider incorporating resistance trends from %R within the institution for increases in AU and adjusting SAARs accordingly. Comprehension of the relationship between %R and SAAR can aid facilities with stewardship programs and understanding how resistance contributes to antibiotic usage.

**Disclosures:**

**Julia Moody, MS**, **Medline** (Other Financial or Material Support, Conducted studies in which participating hospitals received contributed antiseptic product)**Molnlycke** (Other Financial or Material Support, Conducted studies in which participating hospitals received contributed antiseptic product)

